# Linkage Map Development by EST-SSR Markers and QTL Analysis for Inflorescence and Leaf Traits in Chrysanthemum (*Chrysanthemum morifolium* Ramat.)

**DOI:** 10.3390/plants9101342

**Published:** 2020-10-11

**Authors:** Min Fan, Yike Gao, Zhiping Wu, Qixiang Zhang

**Affiliations:** Beijing Key Laboratory of Ornamental Plants Germplasm Innovation and Molecular Breeding, National Engineering Research Centre for Floriculture, School of Landscape Architecture, Beijing Forestry University, Beijing 100083, China; qffanmin@163.com (M.F.); Wuzhiping288@163.com (Z.W.); zqxbjfu@126.com (Q.Z.)

**Keywords:** chrysanthemum, EST-SSR, linkage map, quantitative trait loci (QTLs), inflorescence, leaf traits

## Abstract

Chrysanthemums (*Chrysanthemum morifolium* Ramat.) are famous ornamental crops with high medicinal and industrial values. The inflorescence and leaf traits are key factors that affect the yield and quality of chrysanthemum. However, the genetic improvement of those traits is slow within chrysanthemum because of its hexaploidy, high heterozygosity and enormous genome. To study the genetic control of the important traits and facilitate marker-assisted selection (MAS) in chrysanthemum, it is desirable to populate the genetic maps with an abundance of transferrable markers such as microsatellites (SSRs). A genetic map was constructed with expressed sequence tag–simple sequence repeat (EST-SSR) markers in an F_1_ progeny of 192 offspring. A total of 1000 alleles were generated from 223 EST-SSR primer pairs. The preliminary maternal and paternal maps consisted of 265 marker alleles arranged into 49 and 53 linkage groups (LGs), respectively. The recombined parental maps covered 906.3 and 970.1 cM of the genome, respectively. Finally, 264 polymorphic loci were allocated to nine LGs. The integrated map spanned 954.5 cM in length with an average genetic distance of 3.6 cM between two neighbouring loci. Quantitative trait loci (QTLs) analysis was performed using the integrated map for inflorescence diameter (ID), central disc flower diameter (CDFD), number of whorls of ray florets (NWRF), number of ray florets (NRF), number of disc florets (NDF), number of florets (NF), ray floret length (RFL), ray floret width (RFW), ray floret length/width (RFL/W), leaf length (LL), leaf width (LW) and leaf length/width (LL/W). Overall, 36 (21 major) QTLs were identified. The successful mapping of inflorescence and leaf traits QTL demonstrated the utility of the new integrated linkage map. This study is the first report of a genetic map based on EST-SSR markers in chrysanthemum. The EST-SSR markers, genetic map and QTLs reported here could be valuable resources in implementing MAS for chrysanthemums in breeding programs.

## 1. Introduction

Chrysanthemum (*Chrysanthemum morifolium* Ramat.) belongs to the family Asteraceae and is a well-known ornamental and medicinal crop throughout the world. They are cultivated as cut flowers and potted or garden plants, which occupy a very important position in the global flower industry. It also possesses diverse medicinal properties, including antibacterial, antioxidant, anti-inflammation, anticancer and cardiovascular protection [1]. The dried capitulum of chrysanthemum is a valuable herbal medicine, which can be used for scattering cold, cleaning heat and toxin as well as brightening eyes [2]. It is also used as tea and seasoning, as it is abundant in bioactive compounds, such as polyphenols and flavonols. Nevertheless, linkage analysis and marker-assisted selection (MAS) breeding in chrysanthemum have been challenging due to the hexaploidy (2n = 6x = 54), high heterozygosity, huge genome as well as self-incompatible and outcrossing nature [3]. Although the genome information of *C. nankingense* [4] and *C. seticuspe* [5] has been published, the genomic information of cultivated chrysanthemum has not been reported. Several hypotheses have been proposed in previous reports regarding the ploidy of chrysanthemum, which has been characterized as allopolyploid [6,7], segmental allohexaploid [8,9] and autohexaploid [10]. However, clear conclusions about the ploidy type of chrysanthemum have not yet been reached.

Genetic and quantitative trait loci (QTL) mapping is a powerful approach to identify the genomic regions to control the important traits [11]. That method can greatly enhance the efficiency and precision of conventional breeding. However, to achieve the linkage mapping in polyploids is very difficult. A commonly used approach is to use single-dose (SD) markers combined with pseudotestcross approach, which has been used to construct the genetic maps in several polyploid species, including strawberry [12], sweetpotato [13] and sugarcane [14]. Linkage and quantitative trait loci (QTL) mapping in chrysanthemum is at a preliminary stage. The previous linkage maps in chrysanthemum have been constructed mainly using random amplified polymorphic DNA (RAPD), intersimple sequence repeats (ISSR), amplified fragment length polymorphism (AFLP) markers, sequence-related amplified polymorphism (SRAP) and single nucleotide polymorphism (SNP) markers [15,16,17,18,19,20,21]. In short, most previous genetic maps have been constructed with dominant markers, which are difficult to transfer from one mapping population to another. 

Expressed sequence tag–simple sequence repeat (EST-SSR) markers are defined by specific primer pairs, which are transferable from one progeny to another, even among distantly related species. Therefore, EST-SSR markers are handy tools for map comparison and consensus. Moreover, they may be directly associated with the candidate genes and can be exploited as anchor markers for comparative mapping and evolutionary studies [22], which has been a successful approach in a few species, such as carnation [23] and tea plant [24]. The multiallelic nature of EST-SSR markers also make them more likely to tag a haplotype (or trait) than SNPs in polyploid species [25]. However, in recent years, the researchers have increasingly turned to rely on SNP markers for genotyping. Sequencing data requires greater curation and bioinformatics skills and it may contain more erroneous and missing data, and the tools they used may be not suitable for polyploid datasets [26]. Hence, EST-SSR marker technology remains important.

Although EST-SSR markers have been employed for linkage map construction in many ornamental crops [23,27,28], few studies have been conducted in chrysanthemums. The inflorescence is the main ornamental and medicinal part for chrysanthemum. The leaf traits not only affect the quality of the flowers but also contribute to the overall appearance of the plant. In this study, the genetic linkage map of chrysanthemum was developed based on EST-SSR markers. The obtained genetic map was used to perform QTL analyses of the inflorescence and leaf traits. 

## 2. Results

### 2.1. EST-SSR Marker Segregation 

Among the 262 EST-SSR primer pairs, 223 (85%) were informative and amplified alleles around the expected size, which were used for genotyping the mapping population (Table S1). As a result, a total of 1000 alleles were scored in two parents and 192 F_1_ individuals, among which 285 were paternal-specific, 271 were maternal-specific and 444 were shared. The classification of SSR alleles based on their segregation ratios is listed in Table 1. Of these, 94 alleles (9.4%) were monomorphic in the progeny. The dose of the 906 polymorphic alleles was estimated by χ^2^ test (α = 0.01) to the appropriate expected segregation ratios of hexasomic (random pairing) and disomic (preferential pairing) inheritance, respectively. Among the 906 alleles, 362 uniparental (183 paternal-specific and 179 maternal-specific) and 94 biparental simplex marker alleles were not affected by the inheritance mode, segregating in a 1:1 and 3:1 ratio, respectively. 

Under the hypothesis of hexasomic inheritance, 87 and 38 uniparental SSR alleles gave fits to 4:1 and 19:1 segregation ratios expected for duplex and triplex marker alleles, respectively. In case of biparental SSR marker alleles, while 79 SSR marker alleles gave fits to 9:1 segregation ratio’s expected for duplex-simplex alleles, the dose of 158 alleles could not be identified owing to the multiple fits to different types of segregation ratios. And 88 SSR alleles showed distorted segregation at α < 0.01 under this hexasomic inheritance assumption. On the other hand, under the hypothesis of disomic inheritance, 56 and 49 uniparental SSR alleles gave fits to 3:1 and 7:1 segregation ratios expected for duplex and triplex marker alleles, respectively. While 45 SSR marker alleles gave fits to 7:1 segregation ratio’s expected for duplex-simplex alleles, the dose of 164 alleles could not be identified owing to the multiple fits to different types of segregation ratios. Furthermore, 136 SSR alleles showed distorted segregation at α < 0.01 under this disomic inheritance assumption (Table 1). 

### 2.2. Linkage Map Construction

Firstly, 265 (37.1%) marker alleles were mapped successfully on the preliminary maternal and paternal maps, respectively (Figure S1a,b) (Table S2). Furthermore, three duplex marker alleles were mapped on the preliminary maternal and paternal maps, respectively, which can be used to identify homologous LGs. The preliminary maternal and paternal maps consisted of 49 and 53 LGs, respectively, which putatively corresponded to the number of chromosomes in chrysanthemum (2n = 6x = 54). Those LGs covered a total length of 1800.89 cM in the maternal parent and 2033.52 cM in the paternal parent and had a mean chromosome length of 36.75 and 38.37 cM, respectively. The LGs ranged in size from a low of 2.64 (M49) to a high of 99.44 cM (M1) in the female parent and from 1.06 (F53) to 122.72 (F1) in the male parent (Figure S1a,b) (Table S2). 

Using a set of common SSR markers among LGs, the preliminary maps were mapped to generate recombined parental maps. A total of 270 EST-SSR markers were placed successfully when the two parental maps were considered in step 2, among these markers, 141 originated from female parent and 129 originated from male parent. The recombined map consisted of 10 homologous groups for female and male parent, respectively (Figure S2a,b) (Table S3). The map lengths were 906.28 cM and 970.13 cM, with a mean distance between markers of 6.43 and 7.52 cM for female and male parent, respectively (Table S3). 

**Figure 1:**
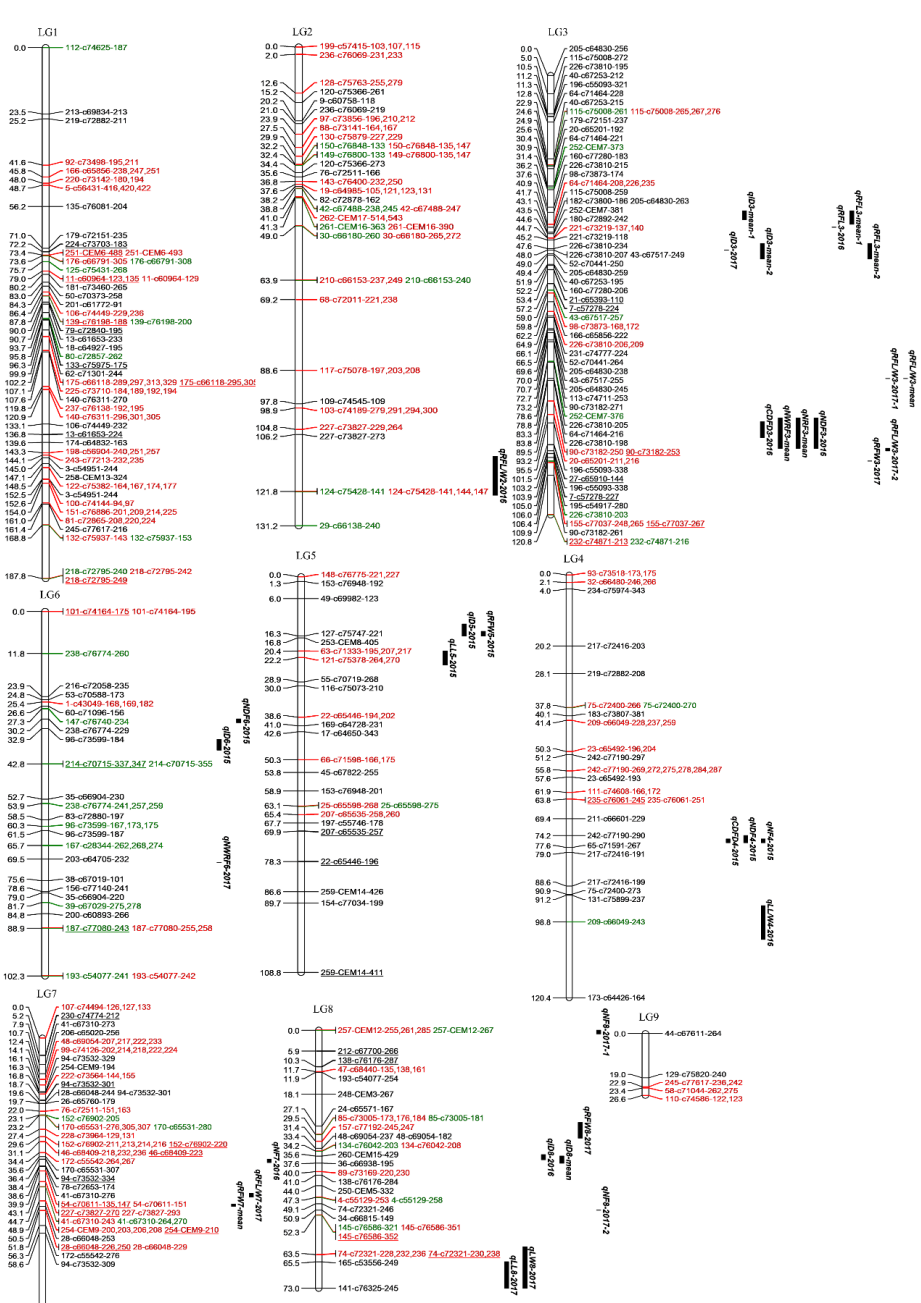
Final integrated map derived from an F_1_ population of 192 offspring in chrysanthemum. Map distances (cM) and SSR marker alleles are shown on the left and right side of each linkage group, respectively. Duplex markers are marked in green. Bridge markers are marked in red. Distorted segregating markers are underlined, which are indicated by a significant *p* value in the chi-squared test: *p* < 0.01; Quantitative trait loci are located on the far right of each LG.

The two recombined maps were combined to form a single integrated map. The final map was composed of 264 loci amplified from 187 polymorphic primer pairs, which formed nine LGs with a total length of 954.46 cM (Figure 1) (Table 2). Nevertheless, the alleles were unevenly distributed, with the number of alleles ranging from 5 (LG 9) to 58 (LG 3) and the mean distance between markers varied from 2.08 (LG3) to 5.32 (LG 9) cM (Table 2). 

### 2.3. Phenotypic Evaluation 

**Figure 2:**
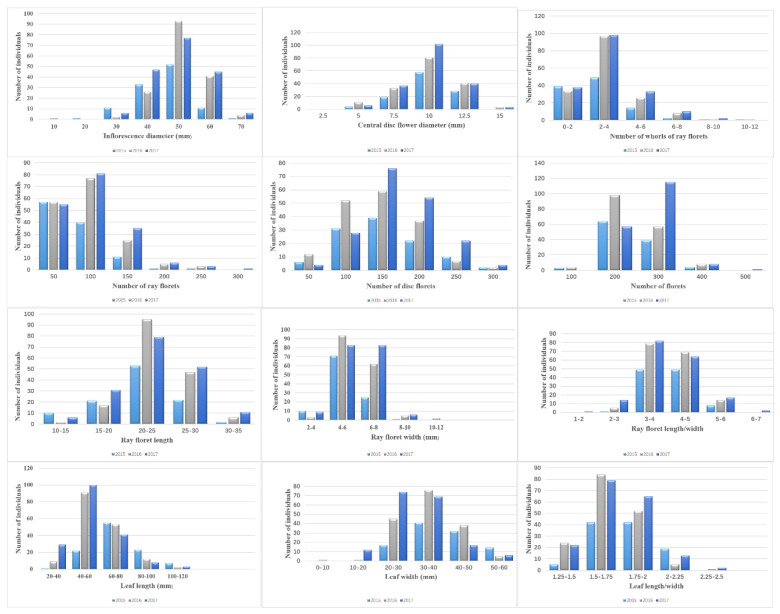
Frequency distributions of 12 inflorescence and leaf traits for the F_1_ progeny in 2015, 2016 and 2017.

The descriptive statistics for inflorescence and leaf traits are listed in Table 3. The frequency distributions of phenotypic values for 12 traits are shown in Figure 2. As some offspring cannot bloom in outdoor conditions, not always all 192 offspring were used. Significant differences between parents were observed for all those traits in at least one year (*p* < 0.01) except RFL/W, LW and LL/W. The correlation analysis revealed that most of the traits were correlated with each other, and a strong degree of correlation was observed between some traits. Significant positive correlations (correlation coefficient > 0.5) were observed between ID and RFL, ID and RFW, CDFD and NDF, NDF and NF, NRF and NWRF, RFL and RFW as well as LL and LW (Table S4). Significant negative correlations (correlation coefficient < −0.5) were observed between CDFD and NWRF (Table S4). Broad-sense heritability of the 12 traits ranged from 0.76 (LW) to 0.95 (NRF). Except for NRF, transgressive genotypes existed for other traits.

### 2.4. QTL Identification

After permutation test, regions with a logarithm of odds (LOD) score of 3.6 were considered as candidate QTL intervals. Overall, 36 QTLs (21 major) were identified (Figure 1) (Table 4). In 2015, eight QTLs (all major) were identified; in 2016, seven QTLs (all major) were found; in 2017, 11 (2 major) QTLs were detected; and 10 (four major) were identified for average data. The PVE ranged from 6.8 to 18.9%.

#### 2.4.1. Inflorescence Traits

Seven and two QTLs were identified to influence ID and CDFD, respectively (Table 4). The *qID8-2016* and *qID8-mean* were colocalized at the same position on LG 8, with overlapping LOD confidence intervals. Interestingly, *qID3-mean-1* and *qRFL3-mean-1* were colocalized at the same position on LG 3. Furthermore, the *qID3-mean-2*, *qRFL3-2016* and *qRFL3-mean-2* were colocalized at another same position on LG 3. In addition, the *qCDFD4-2015* was mapped on the SSR locus 242-c77190-290 on LG 4 where *qNDF4-2015* and *qNF4-2015* were also identified.

For NWRF, NRF, NDF and NF, two, one, three and four QTLs were identified, respectively. Interestingly, the *qNWRF3-mean*, *qNRF3-mean* and *qNDF3-2016* were mapped between the SSR locus 196-c55093-348 and 27-c65910-144 on LG 3, with overlapping LOD confidence intervals. 

A total of three, one and three QTLs were detected to influence RFL, RFW and RFL/W. Interestingly, *qRFL3-2016* and *qRFL3-mean-2* were colocalized at the same position on LG 3, with overlapping LOD confidence intervals. In addition, *qRFL/W3-2017-1* and *qRFL/W3-mean* were mapped on the SSR locus 64-c71464-216 on LG 3, with overlapping LOD confidence intervals.

#### 2.4.2. Leaf Traits

A total of two, one and one QTLs were detected to influence LL, LW, LL/W, respectively (Table 4). The *qLL8-2017* and *qLW8-2017* were colocalized at the same position on LG 8.

## 3. Discussion

In summary, we have developed an integrated linkage map for chrysanthemum using EST-SSR markers, which spanned 954.5 cM in length with an average genetic distance of 3.6 cM between two neighbouring loci. Moreover, 36 (21 major) QTLs were identified for 12 inflorescence and leaf traits. Besides that, it is expected to provide valuable anchor markers to integrate information from future genetic maps. The SSR markers, genetic map and QTL reported here could be valuable resources in chrysanthemum breeding.

### 3.1. EST-SSR Markers

Most SSR primer pairs (190 over 223) provided multilocus amplification in chrysanthemum, which can result from the duplication of zones of genome during the evolution in chrysanthemums, which was also observed in other polyploid species, such as alfalfa [29], *Zoysia matrella* [30] and rose [28]. Besides, EST-SSR markers were derived from the conserved expressed regions of the genome, and thus had greater potential for finding associations with functional genes. In the future, researchers can transfer the EST-SSR markers mapped in this study to other population in the genus *Chrysanthemum*.

### 3.2. Genetic Map Construction

Using 223 EST-SSR primer pairs, the first moderately saturated EST-SSR-based integrated genetic map was constructed in chrysanthemums. A mean interval between the markers on the map reached 3.6 cM, which can meet the requirements of QTL mapping. Due to the homogeneity of chromosome segment between the parents and inadequate markers used, the gaps within the LGs were inevitable. An existence of minor LGs (triplets and doublets) and a number of unlinked markers in the preliminary parental maps indicated that there are several large gaps with few markers [31]. In the integrated map, the gaps larger than 20 cM were observed in LG 1, LG 4 and LG 7. Hence, in order to fill those gaps and increase the density, more markers (SNP markers and SSR markers) are needed to saturate the map. 

### 3.3. Phenotypic Characterization

For the progeny used in this study, there are many traits showing substantial variation. Therefore, such opportunity was used to obtain a comprehensive understanding of the inflorescence and leaf traits. The skewness and kurtosis values of 12 traits indicated that the segregation pattern, in most cases, fitted a normal distribution model approximately which was suitable for QTL identification. One reason for the abnormal distribution of some phenotypic trait in 2015 might be the absence of a few individuals’ phenotypic data. Phenotypic trait distributions of NRF were less uniform than the other traits, especially in 2015 and 2016 (Figure 2). 

The phenotypic correlations among 12 traits suggested a stable association between them in this study. A relatively high heritability (>0.50) for 12 traits were observed (Table 3), which is consistent with the results in Song et al. (2018) [32]. For example, the heritability of NWRF was 0.93 in this study and the value was 0.81 in Song et al. (2018). Those results might indicate that genetic composition plays a major role in determining the inflorescence and leaf traits. There are many transgressive genotypes compared to the parental lines observed in this study. The reason for transgressive genotypes may be the combination of alleles from both parents with effects in the same direction [33], which was also confirmed by the study on the trait of days to heading in rice that a few genes and their combinations expanded the variability whose parents exhibit similar phenotypes [34].

### 3.4. QTL Mapping 

Identification of QTLs underlying target trait is prerequisite for MAS. Zhang et al. [15] identified four QTLs for ID, NWRF and RFL, respectively. In contrast, seven, two and three QTLs for ID, NWRF and RFL were identified in the present study, respectively. For ID, Zhang et al. [15] identified two QTLs on LG Y1. Similarly, two QTLs on LG 3 (*qID3-mean-1*, *qID3-mean-2*) were identified herein. Van Geest et al. [18] detected two minor QTLs for NRF. In contrast, a major QTL for NRF was identified in this study. The phenomenon demonstrated that QTL mapping had population-specific effects. 

QTLs controlling correlated traits are usually located in the same or close LG regions [35]. Previous studies have reported QTL clusters for inflorescence traits in chrysanthemum [15]. Zhang et al. [17] observed two clusters of QTL for LL and LW. Similarly, a cluster of QTLs for LL and LW was found on LG 8. On LG 3, we found two clusters for ID and RFL, and another cluster for CDFD, NDF, NRF and NWRF. In addition, a cluster was found for CDFD, NDF and NF on LG 4. Those results are consistent with the significant correlation between them. The traits controlled by clusters might be explained by QTL with pleiotropy or a set of closely linked loci [36,37].

## 4. Materials and Method

### 4.1. Plant Materials and DNA Extraction

In 2014, two phenotypically different cultivars were used to obtain an F_1_ population by manual cross pollination. The F_1_ mapping population was formed in 2015 by randomly selecting 192 individuals from a total population of 546 plants, which were then propagated via cuttings. The progeny was maintained in the experimental fields at Xiaotangshan, Beijing Forestry University, Beijing, China (40.0° N 116.3° E).

Genomic DNA was isolated from fresh young leaves with a DNA extraction kit (Demeter Biotech, Beijing, China). DNA quality was checked on 1 % agarose gels. The DNA samples were stored at −80℃. 

### 4.2. Genotyping of Mapping Population

A total of 262 EST-SSR primer pairs were analysed, among which 245 primer pairs were developed from the “Jinbudiao” EST database [38] and 17 primer pairs were reported in [39]. All the SSR primer pairs were labelled with fluorescent dyes, and SSR genotyping was carried out using a three-primer strategy, including a forward/reverse primer labelled with FAM, HEX or TAMRA (Beijing Microread Genetics Co., Ltd, Beijing, China), as detailed in the protocol of Sun et al [40]. 

All the primer pairs were initially screened for polymorphisms among six randomly chosen segregating individuals and the two parental samples. The EST-SSR markers that generated reproducible polymorphisms were then used to screen all the 194 samples (192 F_1_ individuals and two parents).

The EST-SSR products (1 μL) were then analysed on an ABI3730 fluorescent analyser with 0.5 μL Rox-500 HD size standard (Microread) and 8.5 μL Hi-Di formamide. The data were analysed using GeneMapper (version 3.2).

### 4.3. Marker Scoring and Marker Segregation Type 

Each allele of specific primer pairs was read, respectively. The alleles were scored by assigning “1” or “0” for the presence or absence of segregating fragments, respectively. The monomorphic bands in the progeny were excluded from segregation analysis. According to the double pseudotestcross mapping strategy [41], the markers were divided by their presence in the maternal parent, the paternal parent and in both.

The dosage of each marker was determined by analysing the segregation ratios of EST-SSR marker alleles (presence vs. absence) in the mapping population, according to the expected segregation ratio of the two inheritance models, hexasomic (random pairing) and disomic (preferential pairing) (Table 5) [9]. The markers were divided into four groups based on their segregation ratios: (a) Simplex markers that are present in a single copy only in one parent and that segregate in a 1:1 (presence:absence) ratio in the progeny; (b) Duplex markers that are present in one parent in two copies and that segregate in a hexasomic (4:1), or disomic (3:1) ratio; (c) Triplex markers that are present in one parent in three copies and that segregate in a hexasomic (19:1), or disomic (7:1) ratio; and (d) Simplex-simplex markers present in both parents in a single copy that segregate in a 3:1 ratio in the progeny (Table 5). Alleles at higher dosages were not analysed as our progeny was too small for segregation analysis in higher dosage situations. The χ^2^ test (α = 0.01) was performed to analyse the goodness-of-fit to the expected segregation ratios for all markers. If the markers did not fit with the Mendelian segregation ratios, they were defined as segregation distortion.

### 4.4. Linkage Map Construction

The progeny was analysed with a double pseudotestcross mapping strategy [41]. JoinMap 4.0 software [42] was used to construct the linkage maps using the cross pollinator (CP) population type code. The genetic distances between markers in centimorgan (cM) were calculated by Kosambi’s [43] mapping function. Firstly, simplex and simplex-simplex markers were used to construct the framework map for each parent at logarithm of odds (LOD) score of 7.0–10.0. Then, duplex markers were inserted into the framework maps [44]. Afterwards, the module “combine groups for map integration” in JoinMap was used to construct a recombined map for each parent separately. Finally, the two data sets were merged for linkage groups on the basis of a subset of common markers that were present in both recombined parental maps [44]. Linkage groups with fewer than five markers were omitted. The linkage groups were drawn by using graphical package MapChart 2.2 for Windows [45]. 

### 4.5. Phenotyping and Statistical Analysis

Phenotypic data of the parents and the F_1_ progeny were collected during three consecutive flowering seasons (2015, 2016 and 2017). Nine inflorescence traits were investigated, including inflorescence diameter (ID), central disc flower diameter (CDFD), number of whorls of ray florets (NWRF), number of ray florets (NRF), number of disc florets (NDF), number of florets (NF), ray floret length (RFL), ray floret width (RFW) and ray floret length/width (RFL/W). The leaf traits were characterized by the leaf length (LL), leaf width (LW) and leaf length/width (LL/W). They were measured from three samples per plant. Statistical analysis of phenotypic data was conducted using Microsoft Excel 2016 or IBM SPSS Statistic 20.0 software. The difference in the traits between two parents was compared using a t test (*p* < 0.05). Pearson’s phenotypic correlation coefficients between different observations of each trait were calculated using the means of three years, respectively. Variance components of the trait scores were estimated by an analysis of variance (ANOVA) using the general linear model procedure of the statistical software IBM SPSS Statistic 20.0 with years as fixed effects. The results were used to calculate broad sense heritability according to the equation: *h*^2^ = *σ*^2^*g **/*** (*σ*^2^*_g_*+ *σ*^2^*_g_*_∗_*_y_****/*** *m* + *σ*^2^*_e_**/** rm*), where (*σ*^2^*_g_*), (*σ*^2^*_g_*_∗_*_y_/m*) and (*σ*^2^*_e_/rm*) are the genetic, genotype × year interaction, and residual variance components, *m* is the number of years and *r* is the number of replications. In 2015, the data obtained was less than in the other two years due to some individuals’ phenotypic data not being measured. In addition, a dozen chrysanthemums cannot bloom in outdoor conditions every year, which lead to an absence of some individuals’ phenotypic data. The average values of all traits each year were calculated for QTL analysis. 

### 4.6. QTL Analysis 

QTL analysis was performed using MapQTL6.0 [46]. A permutation test (1000 times) was performed with a significance level of 5% to calculate the LOD score as the threshold value for QTL detection. First, interval mapping (IM) was used to find QTL regions associated to each of the traits tested. The markers that were closely linked to the positions with the highest LOD score were taken as cofactors, and tested using the automatic cofactor selection (ACS) procedure, with the P-value cutoff for elimination of a cofactor set at *p* = 0.02. Using the set of cofactors, multiple QTL mapping (MQM) was conducted. According to the percentage of phenotypic variation explained (PVE), QTL with PVE more than 10.0 was classified as major QTL, while that with PVE less than 10.0 was classified as minor QTL. The identified QTL was named with q, followed by a trait abbreviation, a LG number, a hyphen (-) and a number indicating the year of its expression. If two or more QTL were identified for a trait on the same LG in the same year, a hyphen (-) with a serial number was suffixed. For example, *qID1-2017-2* indicates the second QTL underlying ID on LG 1 by analysing the data from 2017. The linkage groups representing QTL were drawn using MapChart 2.2 [45]. 

## Figures and Tables

**Table 1:** Classification of 1000 alleles detected in 223 expressed sequence tag–simple sequence repeat (EST-SSR) loci using the χ^2^ goodness of fit test (α = 0.01, df = 1) depending on the segregation ratios of hexasomic and disomic inheritance.

Marker Types	Autohexaploid (Hexasomic)				Allohexaploid (Disomic)			
	No. of Alleles Present in the Paternal Parent	No. of Alleles Present in the Maternal Parent	No. of Alleles Present in Both Parents	Total	No. of Alleles Present in the Paternal Parent	No. of Alleles Present in the Maternal Parent	No. of Alleles Present in Both Parents	Total
Monomorphic	3	2	89	94	3	2	89	94
Polymorphic								
Simplex × nulliplex	183	179	-	362	183	179	-	362
Duplex × nulliplex	50	37	-	87	28	28	-	56
Triplex × nulliplex	23	15	-	38	28	21	-	49
Simplex × simplex	-	-	94	94	-	-	94	94
Duplex × simplex			79	79	-	-	45	45
Unidentified dose	0	0	158	158	8	6	150	164
Distorted at α < 0.01	26	38	24	88	35	35	66	136
Total	285	271	444	1000	285	271	444	1000

**Table 2:** Distribution of markers on the final integrated map and linkage group statistics.

Linkage Group	Number of SSR Marker Alleles	Duplex Markers	Number of Markers Exhibiting Segregation Distortion (*p* < 0.01)	Map Length (cM)	Map Density (loci/cM)	Average Distance Between Markers (cM)	Largest Gap Between Markers (cM)
LG1	45	3	2	187.80	0.24	4.17	23.49
LG2	29	1	0	131.22	0.22	4.52	19.44
LG3	58	4	2	120.79	0.48	2.08	10.92
LG4	23	1	0	120.39	0.19	5.23	21.64
LG5	23	0	3	108.81	0.21	4.73	19.15
LG6	24	1	0	102.32	0.23	4.26	13.43
LG7	33	1	2	83.48	0.40	2.53	24.92
LG8	24	0	1	73.04	0.33	3.04	11.16
LG9	5	0	0	26.61	0.19	5.32	18.96
Total	264	11	10	954.46	0.28	3.62	24.92

**Table 3:** Parent mean (± SD) and population mean (± SD) and range for 12 inflorescence and leaf traits. Skewness and kurtosis (± SE) and values of broad-sense heritability for 12 traits are also listed.

Trait	Trait Code	Year	Parent Mean ± SD		Significant Mean Difference Among Parental Values (*t*-Test)	F_1_ Population Mean ± SD	Range	Skewness	Kurtosis	Heritability
			F	M						
Inflorescence diameter	ID	2015	44.52 ± 2.2	45.79 ± 2.84	n.s	40.57 ± 7.95	16.68–60.7	−0.32	0.23	0.81
		2016	63.26 ± 1.42	47.62 ± 5.16	Yes, *p* < 0.001	45.86 ± 7.42	6.20–63.27	−1.04	4.59	
		2017	59.98 ± 2.11	41.09 ± 2.94	Yes, *p* < 0.01	45.01 ± 8.58	20.06–65.86	−0.01	−0.45	
Central disc flower diameter	CDFD	2015	10 ± 0.75	3.03 ± 0.80	Yes, *p* < 0.01	8.80 ± 1.69	3.95–12.05	−0.39	−0.18	0.90
		2016	10.16 ± 0.84	3.32 ± 0.46	Yes, *p* < 0.001	8.58 ± 1.99	2.92–14.33	−0.46	0.61	
		2017	12.79 ± 0.49	3.5 ± 0.28	Yes, *p* < 0.001	8.72 ± 1.71	4.11–12.95	−0.15	−0.07	
Number of whorls of ray florets	NWRF	2015	1 ± 0	9.67 ± 1.53	Yes, *p* < 0.05	2.83 ± 1.44	0–8.5	1.20	2.43	0.93
		2016	1 ± 0	8.5 ± 0.50	Yes, *p* < 0.05	3.28 ± 1.52	1–12.67	1.95	7.98	
		2017	1 ± 0	8.67 ± 0.58	Yes, *p* < 0.05	3.32 ± 1.44	1–8.33	0.95	0.63	
Number of ray florets	NRF	2015	14.33 ± 0.58	209.67 ± 43.11	Yes, *p* < 0.01	60.01 ± 31.60	13–173	1.31	1.45	0.95
		2016	13.33 ± 1.15	239 ± 14.11	Yes, *p* < 0.001	70.71 ± 38.5	17.67–245.67	1.52	3.34	
		2017	16 ± 2.00	297.33 ± 25.17	Yes, *p* < 0.001	76.51 ± 40.34	21–213.33	1.02	0.72	
Number of disc florets	NDF	2015	118 ± 14.73	16.67 ± 3.06	Yes, *p* < 0.01	131.28 ± 54.40	12–274.33	0.41	−0.08	0.88
		2016	92.33 ± 10.50	17.67 ± 6.03	Yes, *p* < 0.001	121.56 ± 49.96	16–292	0.43	0.32	
		2017	226 ± 6.00	29 ± 6.56	Yes, *p* < 0.001	144.53 ± 46.03	41.33–265.33	0.27	−0.24	
Number of florets	NF	2015	132.33 ± 15.28	226.33 ± 40.80	n.s	191.67 ± 55.74	53.00–355	0.46	0.16	0.85
		2016	105.67 ± 11.50	256.67 ± 20.13	Yes, *p* < 0.001	192.41 ± 57.35	95.67–396.33	0.66	0.39	
		2017	242 ± 8.00	326.33 ± 30.11	Yes, *p* < 0.01	221.57 ± 42.22	122.67–421.5	0.82	2.23	
Ray floret length	RFL	2015	26.47 ± 2.02	21.18 ± 1.54	Yes, *p* < 0.05	21.86 ± 4.23	13.17–33.9	−0.05	0.02	0.83
		2016	34.77 ± 0.62	21.63 ± 3.55	Yes, *p* < 0.05	23.56 ± 3.54	10.51–32.54	−0.22	1.00	
		2017	32.7 ± 0.25	21.52 ± 0.83	Yes, *p* < 0.05	23.15 ± 4.48	11.12–35.79	0.16	0.06	
Ray floret width	RFW	2015	7 ± 0.58	5.40 ± 0.43	Yes, *p* < 0.05	5.39 ± 0.99	2.9–8.61	−0.01	0.76	0.86
		2016	8.14 ± 1.12	4.75 ± 0.30	Yes, *p* < 0.05	5.97 ± 1.32	3.2–15.42	2.81	16.57	
		2017	7.97 ± 0.30	4.91 ± 0.05	Yes, *p* < 0.05	5.90 ± 1.17	2.36–9.06	−0.24	0.25	
Ray floret length/width	RFL/W	2015	3.78 ± 0.08	3.93 ± 0.37	n.s	4.12 ± 0.67	2.98–7.52	1.30	4.90	0.90
		2016	4.32 ± 0.55	4.54 ± 0.50	n.s	4.06 ± 0.62	2.71–15.42	0.46	−0.04	
		2017	4.11 ± 0.13	4.39 ± 0.20	n.s	4.04 ± 0.87	1.88–9.50	1.69	7.92	
Leaf length	LL	2015	44.05 ± 0.58	60.07 ± 7.49	Yes, *p* < 0.05	71.94 ± 15.95	39.95–111.21	0.45	−0.15	0.78
		2016	46.15 ± 4.83	58.04 ± 3.59	Yes, *p* < 0.05	58.93 ± 14.13	22.47–110.24	0.75	1.13	
		2017	45.91 ± 3.13	51.43 ± 4.74	n.s	54.25 ± 15.31	23.82–110.59	1.04	1.80	
Leaf width	LW	2015	26.11 ± 0.45	36.69 ± 8.43	n.s	40.48 ± 9.26	24.1–63.8	0.51	−0.25	0.76
		2016	30.28 ± 3.34	33.52 ± 3.30	n.s	35.18 ± 8.35	6.22–61.47	0.33	0.63	
		2017	28.48 ± 3.34	30.43 ± 2.64	n.s	31.82 ± 9.73	15.77–77	1.54	4.24	
Leaf length/width	LL/W	2015	1.69 ± 0.05	1.66 ± 0.17	n.s	1.80 ± 0.20	1.32–2.23	0.00	−0.31	0.82
		2016	1.53 ± 0.04	1.74 ± 0.15	n.s	1.70 ± 8.35	1.24–3.84	4.09	32.89	
		2017	1.62 ± 0.14	1.69 ± 0.07	n.s	1.73 ± 0.20	1.31–2.37	0.33	−0.01	

*n.s.* nonsignificant.

**Table 4:** Characterization of QTLs for inflorescence and leaf traits in chrysanthemum.

Traits	QTL	Year	Linkage Group	Marker Interval	QTL Position (cM)	Max LOD Value	Contributions (%)
Inflorescence diameter	*qID5-2015*	2015	5	127-c75747-221	13.968	3.69	14.7
	*qID6-2015*	2015	6	96-c73599-184	37.884	3.87	15.3
	*qID8-2016*	2016	8	260-CEM15-429	35.602	4.41	12.4
	*qID3-2017*	2017	3	226-c73810-207; 43-c67517-249	48.031	3.81	9.3
	*qID3-mean-1*	Mean	3	98-c73873-174	37.579	4.51	10.5
	*qID3-mean-2*	Mean	3	226-c73810-234; 226-c73810-207; 43-c67517-249; 52-c70441-250; 205-c64830-259	49.403	5.45	12.6
	*qID8-mean*	Mean	8	260-CEM15-429; 36-c66938-195	36.602	4.48	9.1
Central disc flower diameter	*qCDFD4-2015*	2015	4	242-c77190-290	75.158	3.6	14.2
	*qCDFD3-2016*	2016	3	196-c55093-348	95.174	4.28	11.9
Number of whorls of ray florets	*qNWRF6-2017*	2017	6	203-c64705-232	70.453	3.71	9.1
	*qNWRF3-mean*	Mean	3	196-c55093-348; 27-c65910-144	99.468	4.04	9.5
Number of ray florets	*qNRF3-mean*	Mean	3	196-c55093-348; 27-c65910-144	67.734	3.19	6.8
Number of disc florets	*qNDF4-2015*	2015	4	242-c77190-290	76.158	4.92	18.9
	*qNDF6-2015*	2015	6	238-c76774-229	30.209	3.69	14.5
	*qNDF3-2016*	2016	3	196-c55093-348; 27-c65910-144	102.478	4.45	12.4
Number of florets	*qNF4-2015*	2015	4	242-c77190-290	76.158	3.79	14.9
	*qNF7-2016*	2016	7	94-c73532-334	36.357	4.03	11.4
	*qNF8-2017-1*	2017	8	257-CEM12-285	0	3.99	9.8
	*qNF8-2017-2*	2017	8	34-c66815-149	50.854	3.63	8.9
Ray floret length	*qRFL3-2016*	2016	3	115-c75008-259	41.748	3.74	10.7
	*qRFL8-mean*	Mean	8	260-CEM15-429	36.602	3.97	9.3
	*qRFL3-mean-1*	Mean	3	98-c73873-174; 64-c71464-208	37.579	4.29	10
	*qRFL3-mean-2*	Mean	3	226-c73810-234; 226-c73810-207; 43-c67517-249; 52-c70441-250; 205-c64830-259	47.975 48.031	5.14 5.14	11.9 11.9
Ray floret width	*qRFW5-2015*	2015	5	127-c75747-221	16.261	3.69	14.9
	*qRFW3-2017*	2017	3	226-c73810-203	106.019	3.98	9.7
	*qRFW8-2017*	2017	8	24-c65571-167; 85-c73005	29.128	4.07	9.9
	*qRFW7-mean*	Mean	7	28-c66048-253	50.477	3.67	8.6
Ray floret length/width	*qRFL/W2-2016*	2016	2	124-c75428-144	117.158	4.4	12.4
	*qRFL/W3-2017-1*	2017	3	64-c71464-216	83.312	3.77	9.2
	*qRFL/W3-2017-2*	2017	3	196-c55093-338	102.478	3.91	9.6
	*qRFL/W7-2017*	2017	7	254-CEM9-208	47.708	3.82	9.4
	*qRFL/W3-mean*	Mean	3	64-c71464-216	83.312	3.65	8.6
Leaf length	*qLL5-2015*	2015	5	63-c71333-217; 121-c75378-264	20.383	3.68	14.8
	*qLL8-2017*	2017	8	165-c53556-249; 141-c76325-245	73.038	4.65	11.3
Leaf width	*qLW8-2017*	2017	8	165-c53556-249; 141-c76325-245	73.038	5	12.1
Leaf length/width	*qLL/W4-2016*	2016	4	209-c66049-243	97.241	5.67	15.7

**Table 5:** Expected segregation ratios for the inheritance of a dominant marker in hexaploid chrysanthemum according to two cytological hypotheses (Park et al. 2015).

Marker Dosage	Hypothesis I	Hypothesis II
	Autohexaploid (Hexasomic)	Allohexaploid (Disomic)
Simplex × nulliplex	1:1	1:1
Duplex × nulliplex	4:1	3:1
		1:0
Triplex × nulliplex	19:1	7:1
		1:0
Simplex × simplex	3:1	3:1
Duplex × simplex	9:1	7:1
Triplex × simplex	39:1	15:1
Duplex × duplex	24:1	15:1

## Supplementary Materials

The following are available online at https://www.mdpi.com/2223-7747/9/10/1342/s1, Figure. S1 (**a**) Preliminary linkage groups of the maternal parent derived from an F_1_ population of 192 offspring in chrysanthemum. Map distances (cM) and SSR marker alleles are shown on the left and right side of each linkage group, respectively. Duplex markers are marked in green. Distorted segregating markers are underlined, which are indicated by a significant *p*-value in the chi-squared test: *p* < 0.01, (**b**) Preliminary linkage groups of the paternal parent derived from an F_1_ population of 192 offspring in chrysanthemum. Map distances (cM) and SSR marker alleles are shown on the left and right side of each linkage group, respectively. Duplex markers are marked in green. Distorted segregating markers are underlined, which are indicated by a significant *p*-value in the chi-squared test: *p* < 0.01; Figure S2. (**a**) Recombined maternal map derived from an F_1_ population of 192 offspring in chrysanthemum. Map distances (cM) and SSR marker alleles are shown on the left and right side of each linkage group, respectively. Bridge markers are marked in red. Duplex markers are marked in green. Distorted segregating markers are underlined, which are indicated by a significant *p*-value in the chi-squared test: *p* < 0.01; (**b**) Recombined paternal map derived from an F_1_ population of 192 offspring in chrysanthemum. Map distances (cM) and SSR marker alleles are shown on the left and right side of each linkage group, respectively. Bridge markers are marked in red. Duplex markers are marked in green. Distorted segregating markers are underlined, which are indicated by a significant *p*-value in the chi-squared test: *p* < 0.01. Table S1. Characteristics of the 223 EST-SSR primer pairs used on the map (248–262 were reported in Liu et al., 2015); Table S2. Distribution of markers on the preliminary maternal and paternal maps and linkage group statistics; Table S3. Distribution of markers on the recombined maternal and paternal maps and linkage group statistics; Table S4. Pearson correlation analysis among inflorescence and leaf traits.
