# Supplementary material for: Linkage Map Development by EST-SSR Markers and QTL Analysis for Inflorescence and Leaf Traits in Chrysanthemum (Chrysanthemum morifolium Ramat.)
Source: Plants (Basel). 2020 Oct 11;9(10):1342. doi: 10.3390/plants9101342 (PMC7600071; doi:10.3390/plants9101342)
Supplement: Supplementary file 1 [file plants-09-01342-s001.zip › Supplementary materials/Table S4.docx]

**Table S4** Pearson correlation analysis among inflorescence and leaf traits

| Traits | LL | LW | LL/W | ID | CDFD | NWRF | RFL | RFW | RFL/W | NRF | NDF | NF |
| --- | --- | --- | --- | --- | --- | --- | --- | --- | --- | --- | --- | --- |
| LL | 1 |  |  |  |  |  |  |  |  |  |  |  |
| LW | 0.91** | 1 |  |  |  |  |  |  |  |  |  |  |
| LL/W | 0.14** | -0.24** | 1 |  |  |  |  |  |  |  |  |  |
| ID | 0.14** | 0.19** | -0.09 | 1 |  |  |  |  |  |  |  |  |
| CDFD | 0.20** | 0.20** | -0.02 | 0.37** | 1 |  |  |  |  |  |  |  |
| NWRF | -0.05 | -0.04 | -0.06 | 0.11* | -0.51** | 1 |  |  |  |  |  |  |
| RFL | 0.18** | 0.21** | -0.04 | 0.89** | 0.37** | 0.04 | 1 |  |  |  |  |  |
| RFW | 0.14** | 0.18** | -0.08 | 0.54** | 0.27** | 0.03 | 0.54** | 1 |  |  |  |  |
| RFL/W | -0.40** | -0.34** | -0.10* | 0.18** | -0.01 | 0.12* | 0.10**,* | 0.14** | 1 |  |  |  |
| NRF | -0.09 | -0.05 | -0.10* | 0.12* | -0.45** | 0.89** | 0.05 | 0.02 | 0.16** | 1 |  |  |
| NDF | 0.16** | 0.16** | -0.01 | 0.26** | 0.80** | -0.39** | 0.26** | 0.20** | 0.13** | -0.32** | 1 |  |
| NF | 0.09 | 0.12* | -0.08 | 0.34** | 0.44** | 0.27** | 0.28** | 0.21** | 0.24** | 0.42** | 0.72** | 1 |

**Significant at *p<*0.01

*Significant at *p<*0.05
