# Supplementary figures and images for: Linkage Map Development by EST-SSR Markers and QTL Analysis for Inflorescence and Leaf Traits in Chrysanthemum (Chrysanthemum morifolium Ramat.)
Source: Plants (Basel). 2020 Oct 11;9(10):1342. doi: 10.3390/plants9101342 (PMC7600071; doi:10.3390/plants9101342)

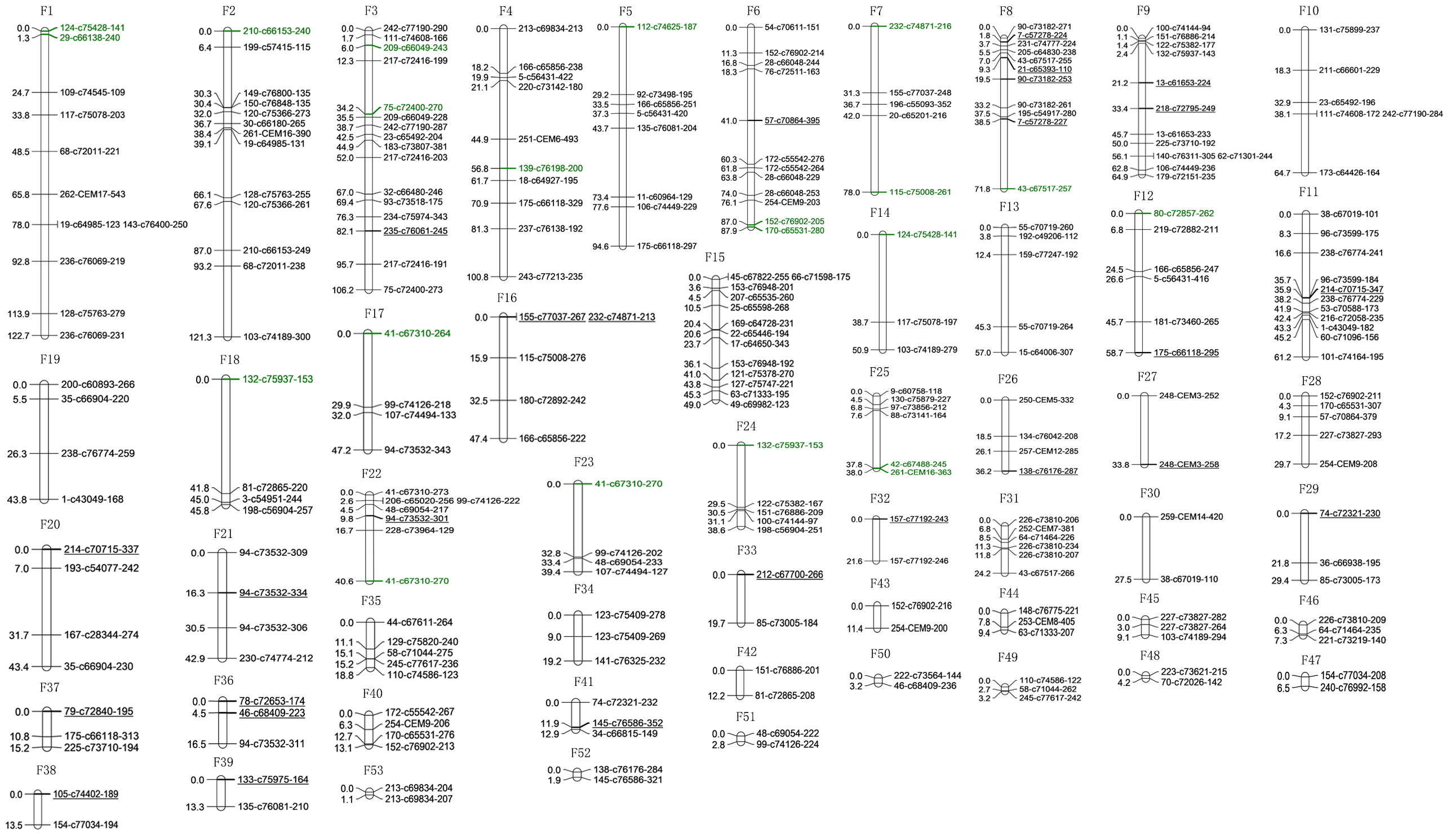

Supplement: Supplementary file 1 [file plants-09-01342-s001.zip › Supplementary materials/Figure S1a.tif]

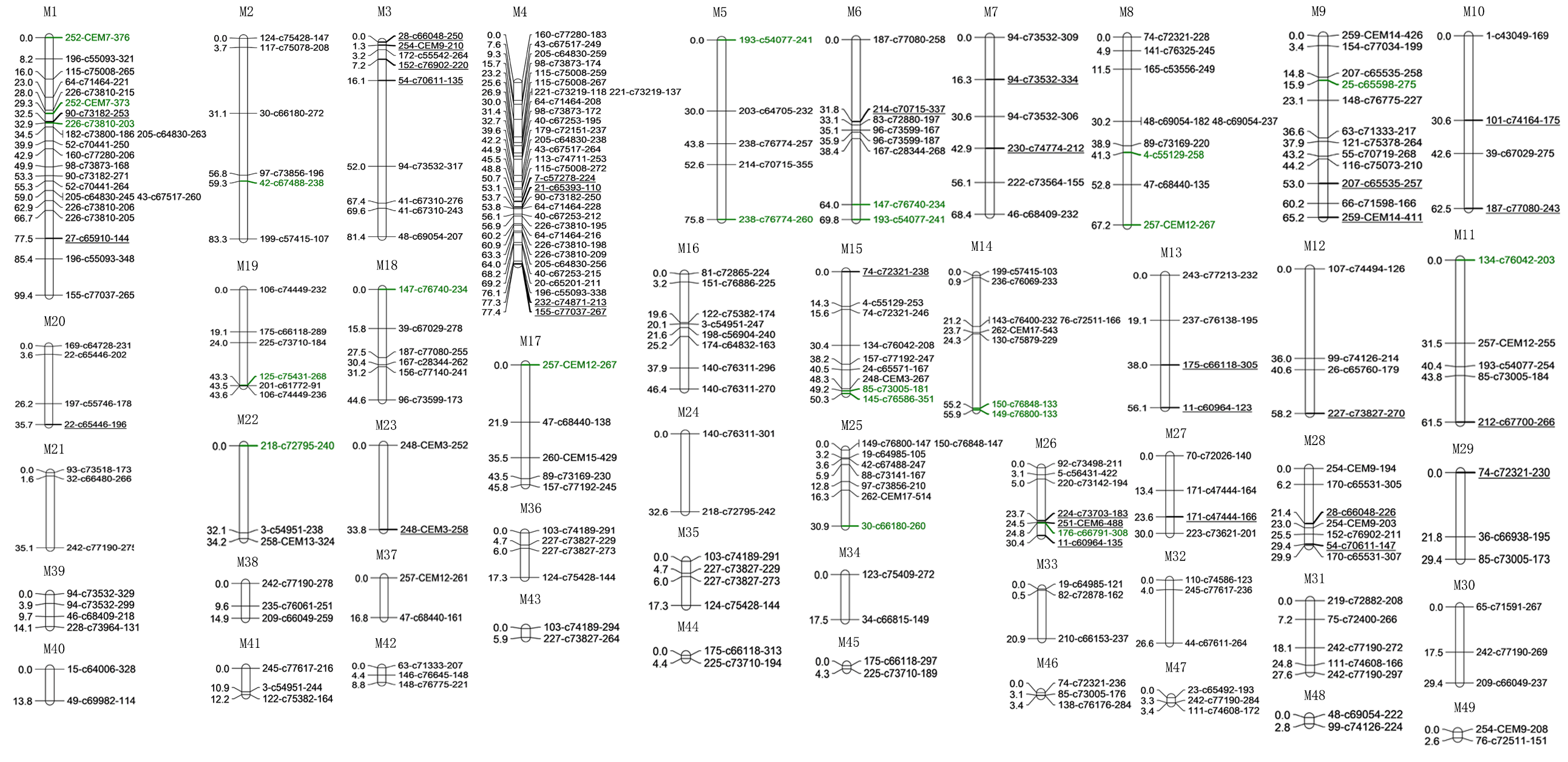

Supplement: Supplementary file 1 [file plants-09-01342-s001.zip › Supplementary materials/Figure S1b.tif]

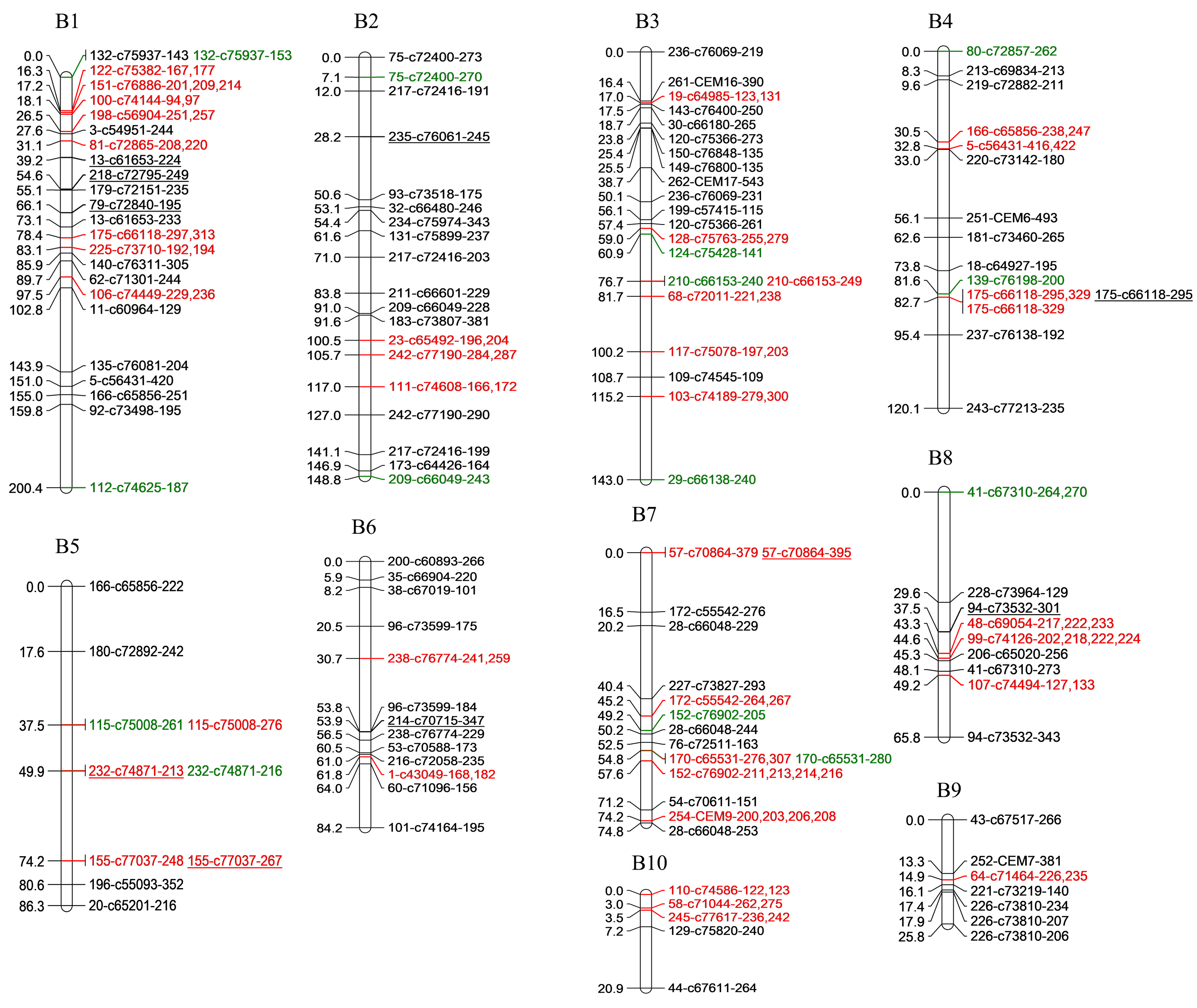

Supplement: Supplementary file 1 [file plants-09-01342-s001.zip › Supplementary materials/Figure S2a.tif]

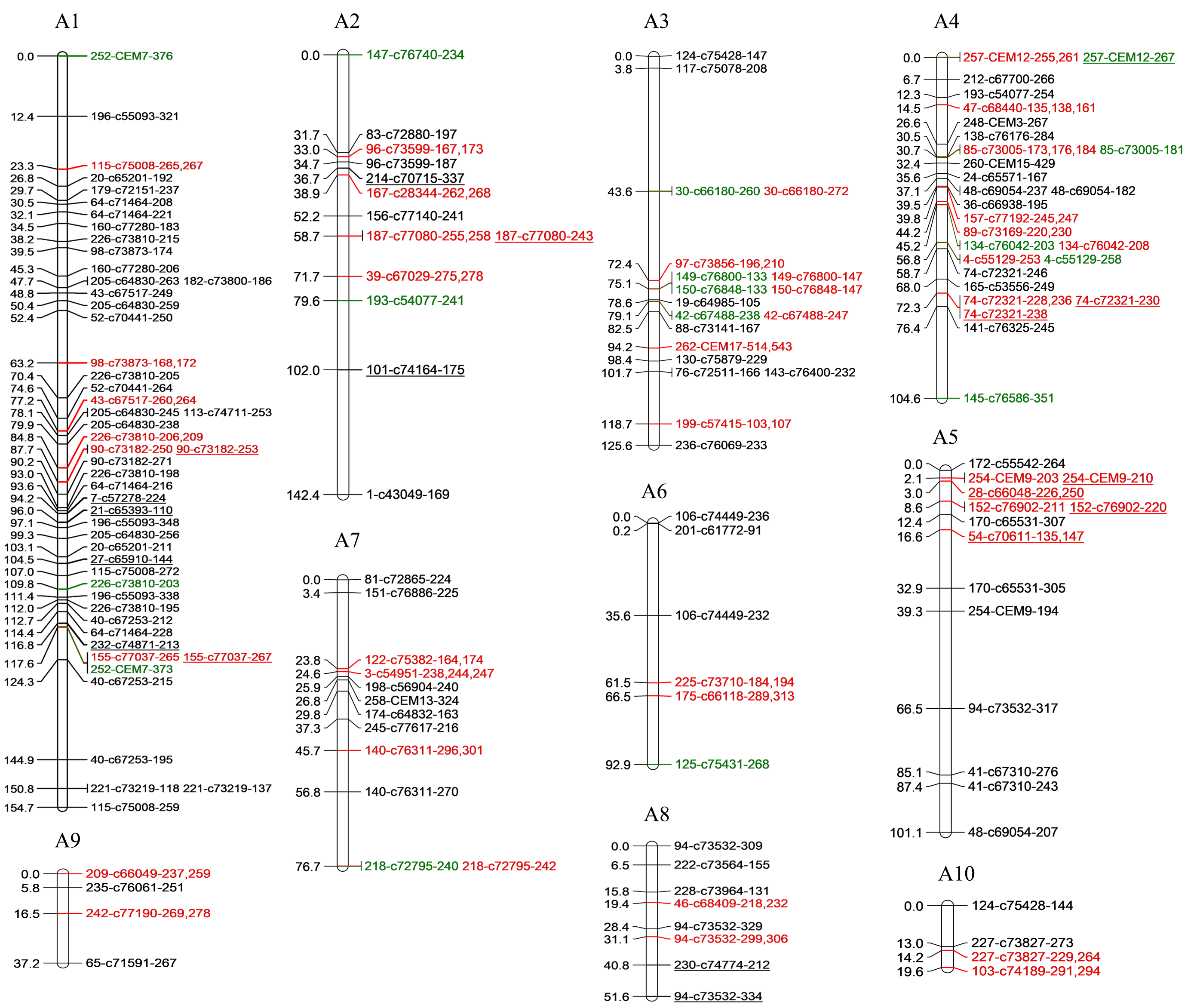

Supplement: Supplementary file 1 [file plants-09-01342-s001.zip › Supplementary materials/Figure S2b.tif]
